# Molecular insertion regulates the donor-acceptor interactions in cocrystals for the design of piezochromic luminescent materials

**DOI:** 10.1038/s41467-021-24381-5

**Published:** 2021-07-02

**Authors:** Chunguang Zhai, Xiu Yin, Shifeng Niu, Mingguang Yao, Shuhe Hu, Jiajun Dong, Yuchen Shang, Zhigang Wang, Quanjun Li, Bertil Sundqvist, Bingbing Liu

**Affiliations:** 1 State Key Laboratory of Superhard Materials, College of Physics, Jilin University, Changchun, China; 2 Institute of Atomic and Molecular Physics, Jilin University, Changchun, China; 3 Department of Physics, Umeå University, Umeå, Sweden

**Keywords:** Optical materials, Organic molecules in materials science, Self-assembly

## Abstract

Developing a universal strategy to design piezochromic luminescent materials with desirable properties remains challenging. Here, we report that insertion of a non-emissive molecule into a donor (perylene) and acceptor (1,2,4,5-tetracyanobezene) binary cocrystal can realize fine manipulation of intermolecular interactions between perylene and 1,2,4,5-tetracyanobezene (TCNB) for desirable piezochromic luminescent properties. A continuous pressure-induced emission enhancement up to 3 GPa and a blue shift from 655 to 619 nm have been observed in perylene-TCNB cocrystals upon THF insertion, in contrast to the red-shifted and quenched emission observed when compressing perylene-TCNB cocrystals and other cocrystals reported earlier. By combining experiment with theory, it is further revealed that the inserted non-emissive THF forms blue-shifting hydrogen bonds with neighboring TCNB molecules and promote a conformation change of perylene molecules upon compression, causing the blue-shifted and enhanced emission. This strategy remains valid when inserting other molecules as non-emissive component into perylene-TCNB cocrystals for abnormal piezochromic luminescent behaviors.

## Abstract (web summary)

The design of piezochromic luminescent materials with desirable stimuliresponsive properties remains challenging. Here, the authors report the insertion of a non-emissive molecule into a donor and acceptor binary cocrystal and realize desirable piezochromic luminescent properties by manipulation of intermolecular interactions between the donor and acceptor molecules.

## Introduction

Luminescent materials that exhibit remarkable changes in emission color and intensity upon external mechanical stimuli, such as pressing, grinding/shearing, and stretching, have been attracting great interest because of their potential for applications in pressure sensors, optical data storage and optoelectronic devices^1–10^. Among these mechanical stimuli, hydrostatic compression is advantageous for a wide range of emission tuning and to build structure–property relationships of materials in a more controllable way^10–14^. So far, most luminescent materials show a gradual red-shifted and quenched emission as pressure increases, which has been explained by different mechanisms, such as exciton coupling^12^, orbital overlap^13^, and *π*–*π* aggregation^15^. In contrast, pressure-induced blue-shifted emission and emission enhancement have only been observed very rarely in luminescent materials upon compression^16^. The design of desirable piezochromic luminescent materials with such anomalous properties for specific applications has long been pursued^9,11^. In particular, *π*-conjugated organic materials, which include a large family of luminescent materials, have been intensively explored^2,5,6^. Very recently, a man-made crystal consisting of the complicated single-component molecule 9-(4-(1,2,2-triphenylvinyl)phenyl)anthracene was found to exhibit novel piezochromic luminescent behavior upon compression^17^. In this case, a discontinuous piezochromic luminescence was observed. First, normal red-shifted and quenched emission was observed at initial compression (up to 1.23 GPa), and then a new photoluminescence (PL) band with blue-shifted and enhanced emission appeared upon further compression to 4.28 GPa. The anomalous luminescent behaviors were explained by a cooperative effect between aggregation-induced emission and energy-transfer suppression.

In contrast to single-component crystals, organic cocrystals (OCCs) are composed of two or more components and the luminescent properties should be more flexible because of their tunable intermolecular interactions and components^8,18–24^. This provides a large number of candidates for studying piezochromic luminescent behaviors and designing new piezochromic luminescent materials with desirable properties. Despite recent efforts, the OCCs reported so far mainly exhibit red-shifted emission and quenched PL upon compression^15,25–27^. Thus, it is important and urgent to develop an innovative and universal strategy for designing piezochromic luminescent materials with desirable pressure-responsive properties, which however, remains challenging.

Here, we report a strategy by inserting an “inert” molecule, tetrahydrofuran, THF, into perylene-1,2,4,5-tetracyanobezene (TCNB) cocrystals (PTCs) for tailoring the donor (perylene)–acceptor (TCNB) interactions and achieve simultaneous pressure-induced blue-shifted and enhanced emission from the cocrystal. In the designed experiment, THF contains saturated *sp*^*3*^-bonded carbon atoms (no *π* electron) and an oxygen atom with paired electrons in *p*/*sp*-orbital, which allows the formation of C–H···O bonding but will not bond covalently with perylene or TCNB. With pressure stabilizing and pushing THF into the PTCs, the interactions between the donor and acceptor (D–A) can be efficiently manipulated. This causes anomalous, simultaneous pressure-induced blue-shifted and enhanced emission in the perylene–TCNB-based cocrystals. This strategy has also been shown to be efficient for other molecules inserted into perylene–TCNB cocrystals as a nonemissive component for anomalous piezochromic luminescence. We also anticipate that it could be extended to other cocrystals with different D–A components, for example, those constructed by aromatic molecules and classical acceptors, such as 7,7,8,8-tetracyanoquinodimethane (TCNQ)^28^ and fullerenes^29^.

## Results

### Characterization of PTCs with and without THF at ambient conditions

**Fig. 1:**
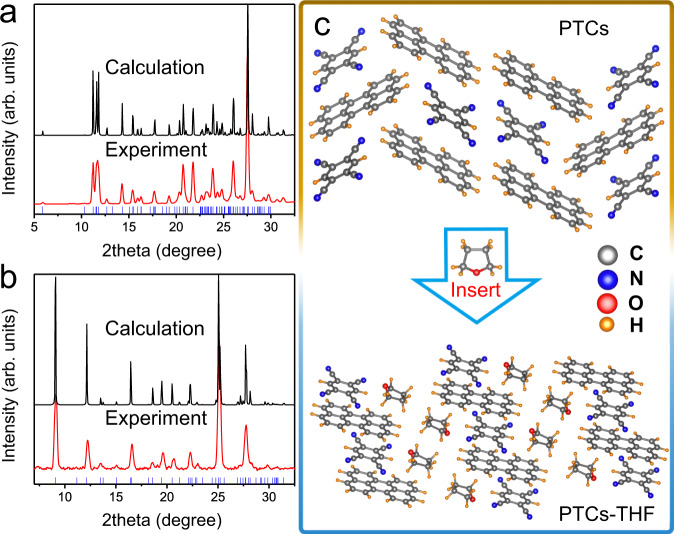
XRD patterns and crystal packing of PTCs and PTCs–THF. Experimental and calculated XRD patterns of **a** PTCs and **b** PTCs–THF. **c** Molecular conformation and crystal packing of PTCs and PTCs–THF at ambient conditions.

Figure 1a and b shows the X-ray diffraction (XRD) patterns of the PTCs and THF-inserted PTCs (PTCs–THF) sealed in two separated glass capillary tubes. Both are in good agreement with the corresponding XRD patterns of our simulated structures. Both the as-prepared PTCs and the PTCs–THF crystallize in a monoclinic structure but with different cell parameters, as summarized in Supplementary Table S1. Their molecular packings in the corresponding crystals are presented in Fig. 1c and Supplementary Figs. S1–S2. In the cocrystals, perylene is a typical polycyclic aromatic hydrocarbon chromophore as the donor (D) component, while TCNB is the acceptor (A). The donor and acceptor molecules are arranged alternately in a similar molecular column (-DADA-), while the neighboring molecular columns are connected to each other, forming a tightly packed stack^11^. For the PTCs, it can be seen that the molecular centers of TCNB and perylene are not above each other, but show a 35% deviation from the accumulation axis^30^; for the PTCs–THF, their center goes back to the same vertical plane. Note that the intercalation of THF molecules would force TCNB to stack toward the edge of perylene. The *π*–*π* overlap between one TCNB molecule and the adjacent perylene molecules is about 50% of a perylene plane^31^.

### Piezochromic luminescent properties of PTCs and PTCs–THF

**Fig. 2:**
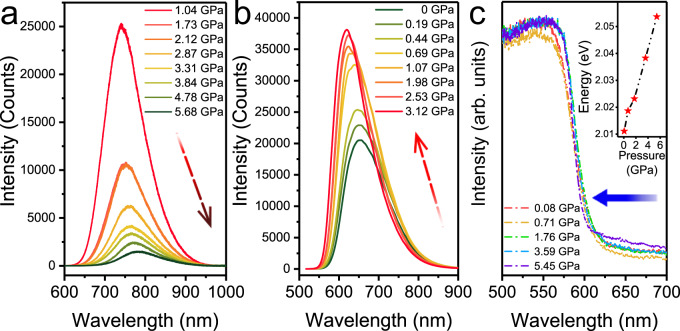
PL and absorption spectra. **a** PL spectra of PTCs upon compression up to 5.68 GPa. **b** PL spectra of PTCs–THF up to 3.12 GPa. **c** In situ UV–Vis absorption spectra of PTCs–THF up to 5.45 GPa. The inset shows the corresponding pressure dependence of the bandgap. The laser powers for the PL measurements of PTCs and PTCs–THF were 17.19 μW and 0.068 μW, respectively.

PL spectra of PTCs upon compression up to 5.68 GPa are shown in Fig. 2a. As we can see from the figure, the PL emission of PTCs shows a normal red shift from 720 to 776 nm as pressure increases, accompanied with an obvious decrease in PL intensity. Such pressure-induced emission changes and fluorescence quenching at certain pressures have also been observed in compression experiments on other cocrystals^15,26^.

To tune the intermolecular interactions between donor and acceptor, THF has been inserted into the lattice of the PTCs to study its effect on the PL emission of the cocrystals. For this, PTCs were soaked in liquid THF in a diamond anvil cell where the blackish-green cocrystals quickly turned red, suggesting that THF molecules penetrated into PTCs, forming PTCs–THF. In this case, THF can be stabilized in the cocrystal by applying pressure and also acts as a pressure-transmission medium surrounding the sample. Remarkably, PTCs–THF exhibits an abnormal PL behavior as pressure increases. As shown in Fig. 2b, the PL emission bands show a clear blue shift from 655 to 619 nm as pressure increases up to 3.12 GPa, accompanied with a significant enhancement of PL intensity. At higher pressure above 3.12 GPa, the PL intensity starts to decrease gradually, but the PL emission bands still keep blue-shifting as pressure increases. Supplementary Fig. S3 demonstrates the piezochromic PL spectra of these two cocrystals up to 20 GPa. To study how the band gap of PTCs–THF changes under pressure, in situ UV–Vis absorption spectra of the material were measured during compression (Fig. 2c). The absorption edge of PTCs–THF is located at 616.6 nm at 0.08 GPa (the corresponding bandgap is 2.01 eV), and exhibits an obvious blue shift during compression. As pressure increases up to 5.45 GPa, the absorption edge of the cocrystal moves to 603.9 nm (corresponding to bandgap 2.06 eV) (inset, Fig. 2c). The band gap of the cocrystal thus increases due to the THF insertion, causing the anomalous emission blue shift as pressure increases. Note that the absorption edge of the cocrystal becomes less sharp and gradually broadens as pressure increases at above 5.45 GPa (Supplementary Fig. S3c), which makes it challenging to derive an accurate bandgap of the compressed cocrystal by curve fitting. This also indicates that interactions in the cocrystal become stronger^32^.

### Structural evolution of PTCs with THF insertion upon compression

**Fig. 3:**
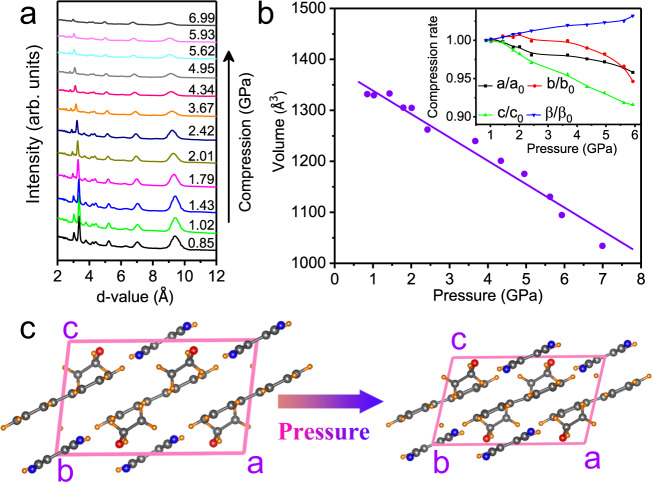
High-pressure XRD patterns of PTCs–THF. **a** High-pressure XRD patterns of PTCs–THF up to 6.99 GPa. **b** The plotted curves for the unit cell volume of PTCs–THF as a function of pressure. The inset shows the compression rate of lattice constants as pressure increases. The XRD patterns are analyzed by JADE. **c** Evolution of the molecular arrangement with increasing pressure (view along the *b*-axis).

To give a further understanding of the effect of THF insertion on the PL emission of our cocrystals upon compression, a high-pressure XRD experiment on PTCs–THF has been performed and the recorded XRD patterns are shown in Fig. 3a. All the diffraction peaks shifted to lower d-values, indicating compression of the lattice. We also present the variation of the unit cell volume with pressure in Fig. 3b. The results suggest that no structural transition happened to PTCs–THF during compression. Instead, the *a-, b-*, and *c-*axes exhibit different pressure evolutions upon compression, indicating an anisotropic compression of the lattice (inset, Fig. 3b). Note that above 3 GPa, the *c-*axis was more compressible than the *a-* and *b-*axes, indicating that the molecules become more parallel and more closely packed in the *ab* plane (Fig. 3c), which could increase the *π*-*π* interactions. Therefore, beyond 3.12 GPa, the reduced distance between D and A in the cocrystal promotes effective *π*–*π* stacking interactions that should be responsible for the emission quenching^33^.

### Intermolecular interactions of PTCs with THF insertion by IR and Raman spectroscopies

**Fig. 4:**
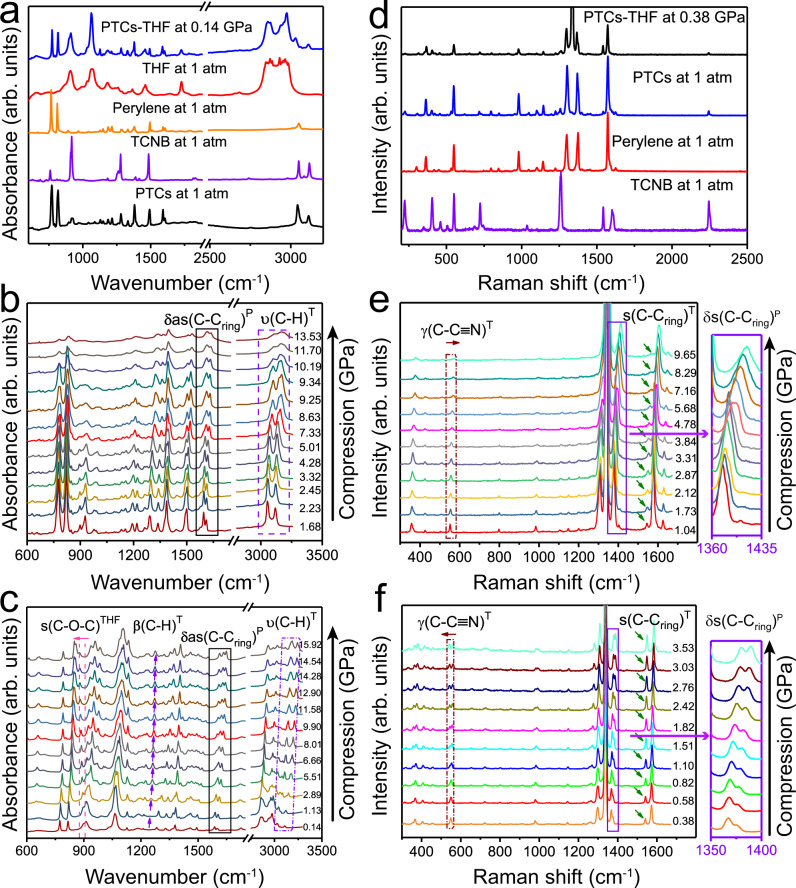
IR and Raman spectra of PTCs and PTCs–THF. **a** The IR spectra of PTCs, THF, and PTCs–THF at/near ambient conditions. High-pressure IR spectra of **b** PTCs and **c** PTCs–THF. **d** Raman spectra of PTCs, TCNB, perylene, and PTCs–THF at/near ambient conditions. High-pressure Raman spectra of the **e** PTCs and **f** PTCs–THF. The relevant vibrations are marked in the diagram, the different marks in the upper right corner represent the vibration attribution; marks *T* and *P* represent TCNB and perylene, respectively.

As no structural transition occurred in the compressed cocrystal, the PL emission of PTCs–THF should be related to the changes of intermolecular interactions in the cocrystals upon compression. These interactions were studied by infrared (IR) spectroscopy. As shown in Fig. 4a, the IR spectra of PTCs, THF, and PTCs–THF were measured. Each of their vibrational modes could be assigned according to our theoretically calculated IR spectra (Supplementary Fig. S4 and table S2). The spectroscopic features from both perylene and TCNB can be clearly distinguished, suggesting only weak van der Waals interactions between the molecules^34^. Upon compression, most of the IR peaks of PTCs were gradually blue-shifted and broadening (Fig. 4b), showing a common pressure evolution as observed in other molecular crystals^25,35,36^. In contrast, PTCs–THF show obvious differences in the IR spectra (Fig. 4c) compared with those of PTCs under pressure, due to the insertion of THF molecules into the lattice. The insertion of THF clearly results in the formation of hydrogen bonding between THF and TCNB. The C–O–C symmetrical stretching vibration s(C–O–C) from THF, located at 895 cm^−1^, exhibited a clear red shift as pressure increased (Supplementary Fig. S5a and b), indicating a strengthening of C–H···O hydrogen bonds. As for TCNB, the formation of C–H···O hydrogen bonds also leads to a much higher blue-shift rate of the C–H stretching vibrations *ν*(C–H) at 3061 and 3108 cm^−1^ in the PTCs–THF than for that in the PTCs upon compression (Supplementary Fig. S5d). A similar effect of the hydrogen bond formation on the blue shift of the C–H stretching mode has also been observed in polyglycine II^37^. In addition, a new IR peak at 1246 cm^−1^, which appears at 1.13 GPa and can be assigned to C–H bending vibrations *β*(C–H) in TCNB^38^, exhibits an obvious enhancement in intensity (Supplementary Fig. S5c), indicating that the polarity of the C–H bond of TCNB increases. This further supports the formation of a blue-shifting hydrogen bond. Consequently, the hydrogen bonding stabilized the TCNB^39–42^. More reasons why the IR peak around 900 cm^−1^ showing a redshift can be assigned to C–O–C vibration and is caused by blue-shifting hydrogen bonds in PTCs–THF are presented in supplementary Fig. S6 and the following notes. On the other hand, the insertion of THF causes a distortion of the perylene molecule. This is evidenced by the gradual asymmetrization and split of the initially asymmetrical deformation vibrations *δ*as(C–C_ring_) of perylene (peak at 1586 cm^−1^) during compression^43^ (Supplementary Fig. S7). The distortion of perylene should reduce the *π*-conjugation^44,45^.

Our Raman measurements give further support for the formation of hydrogen bonding when THF is inserted into the cocrystal upon compression. The recorded Raman spectra at ambient and high pressures are shown in Fig. 4e and f. Each vibrational mode of PTCs and PTCs–THF can be assigned, as shown in Fig. 4d, Supplementary Fig. S8 and Supplementary table 2. The dependence of peak positions and intensities on pressure for some selected Raman modes is shown in Supplementary Fig. S9. It is clear that the relative peak intensity of the carbon ring stretching vibration *ν*(C–C_ring_) at 1541 cm^−1^ becomes stronger as pressure increases from ambient to 3.53 GPa^38^ (Supplementary Fig. S9a, b and S10b), indicating that the polarizability of the C–H bond of TCNB increases^40–42^. This should be related to the enhancement of hydrogen bonding. The Raman peak at 1367 cm^−1^ from symmetrical deformation vibrations *δ*s(C–C_ring_) of perylene gradually splits during compression^43^ (Supplementary Fig. S10a), which indicates the deformation of perylene, in agreement with our IR results. In addition, the peak located at 549 cm^−1^ from C to C≡N out-plane bending vibration *γ*(C–C≡N) of TCNB exhibits a split at 1.1 GPa and one of the split peaks (545 cm^−1^) downshifts to low frequency up to 3.03 GPa^38^ (Supplementary Fig. S9c and d), which indicates that THF restricts the C–C≡N out-of-plane bending vibration (Supplementary Fig. S10c). This could inhibit nonradiative emission and thus promote PL enhancement.

Besides its capability to form hydrogen bonding with TCNB and affect the molecular vibrations, as well as to distort the perylene conformation, the THF inserted into the cocrystal also acts as a spacer to separate and stabilize the TCNB and perylene molecules due to its “inert” properties (the inability to form covalent bonds). Neither Raman nor IR measurements show any obvious weakening or broadening of any IR or Raman peak from TCNB or perylene upon compression, in contrast to the common pressure evolution of weakening and broadening of the corresponding modes in PTCs upon compression (Supplementary Fig. S11).

### Calculation of the molecular orbitals (MO) and photoluminescent properties

**Fig. 5:**
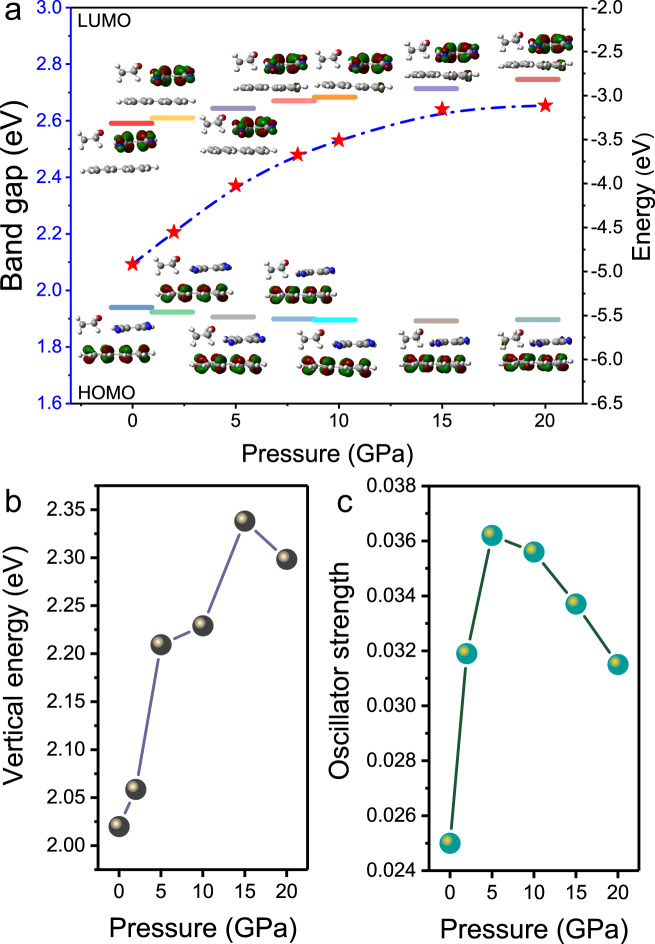
Calculated MO and optical properties. **a** Calculated HOMO–LUMO energy gap (denoted by red star) of PTC–THF from 0 to 20 GPa. The inset shows the distribution of the frontier orbitals at different pressures. The calculated **b** vertical energy and **c** oscillator strength of PTCs–THF from 0 to 20 GPa.

The MO were further calculated to analyze the change of the HOMO–LUMO energy gap^15,19^ (Fig. 5a). Upon THF insertion, the energy gap is increased from 1.836 eV in PTCs to 2.093 eV in PTCs–THF at ambient pressure (Supplementary Fig. S12). The HOMO is distributed mainly on perylene, while the LUMO is distributed mainly on TCNB. Note that THF is not involved in the observed frontier orbital distribution. The distribution of frontier orbitals in the cocrystal does not change obviously, but the energy gap of PTCs–THF increases from 2.093 eV to 2.654 eV upon compression from 0 to 20 GPa. Meanwhile, the vertical energy from our calculation also exhibits a similar pressure evolution as the HOMO–LUMO energy gap and becomes larger as pressure increases (Fig. 5b), which agrees well with the experimentally observed blue-shifted emission. The oscillator strengths of the S_1_ → S_0_ electronic transition were calculated to analyze the change of the PL intensity^46^. The oscillator strength of PTCs–THF is increased from 0.025 to 0.0362 when pressure is increased from 0 to 5 GPa, while it decreases as pressure increases at above 5 GPa (Fig. 5c). These changes in the oscillator strength, suggesting an increase in emission intensity as pressure increases up to 5 GPa and a gradual quenching above 5 GPa, are in very good agreement with our experiments.

### Analysis of noncovalent interactions (NCI) and molecular configurations

**Fig. 6:**
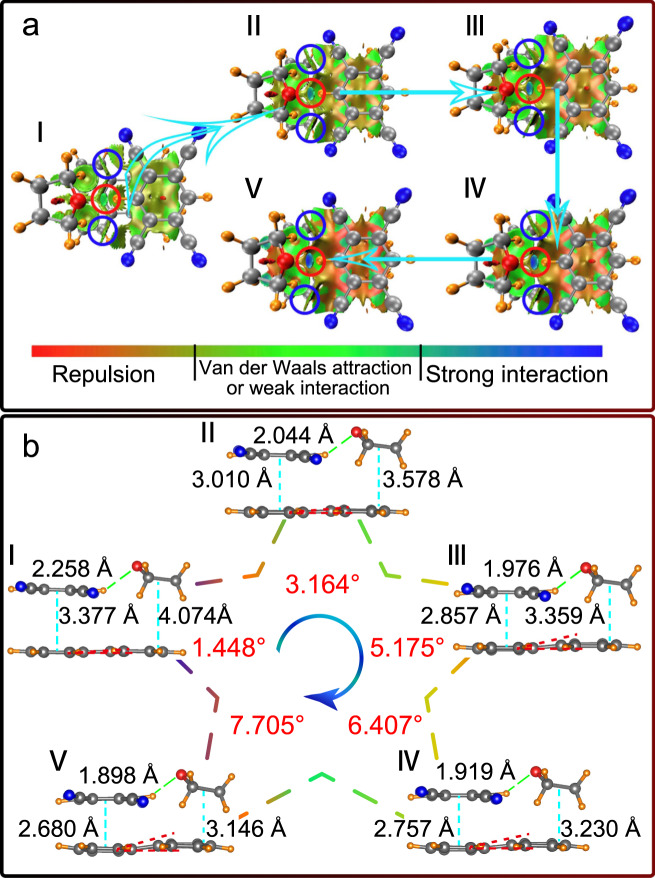
NCI and molecular configurations analysis. **a** NCI analysis of PTC–THF at different pressures. The hydrogen bond strength of C–H···O is highlighted by red circles, while the blue circles indicate that Van der Waals forces still exist between TCNB and THF. **b** The molecular configurations at different pressures. The angle (red number) represents the degree of distortion of perylene from 0 to 20 GPa. Roman numerals represent the calculated structure at 0 GPa (**I**), 5 GPa (**II**), 10 GPa (**III**), 15 GPa (**IV**) and 20 GPa (**V**).

The experimentally observed changes in NCI in the PTCs–THF system have been further studied by the Multiwfn software^47^. As shown in Fig. 6a, the hydrogen bond strength C–H···O increases gradually (color change from dark green to cyan) as pressure increases^48^ (red circles in Fig. 6a). Besides this, the THF insertion also plays a role for isolation and stabilization of TCNB (blue and red circles in Fig. 6a) in the cocrystal upon compression. Moreover, we find that, due to the rigidity of THF molecules, the THF insertion also causes a configurational distortion of the perylene upon compression (see Fig. 6b, the planar perylene turns to a configuration with a twisted angle of 7.705° at 20 GPa). All our theoretical calculations and experiments thus show that the THF insertion significantly affects the molecular configuration of the donor and the acceptor and their intermolecular interactions, and is thus the main reason for the pressure-induced blue shift and emission enhancement by affecting the HOMO–LUMO energy gap.

## Discussion

Our results show that molecular insertion can modify and control the interactions between donor and acceptor in a cocrystal. To examine if this strategy is universal for constructing new piezochromic luminescent materials, some other molecules, such as 1,4-dioxane, pyridine, m-xylene, CCl_4_, benzene, and toluene have also been studied. We found that those molecules analogous to THF, such as 1,4-dioxane and pyridine, are also efficient for achieving photoluminescent materials with anomalous pressure-responsive emission. In these cases, TCNB and perylene act as donor and acceptor, respectively, while the inserted molecule acts as a nonemissive component. Note that pressure-induced blue shift and emission enhancement of fluorescence have been observed in these cocrystals, while their enhancement magnitude, pressure-tuned emission range, as well as transition pressures for emission quenching depend on the inserted molecules. In contrast, CCl_4_ and m-xylene cannot be inserted into PTCs (Supplementary Fig. S13). The results suggest that our strategy can be extended to other molecules for various piezochromic luminescent behaviors based on perylene–TCNB cocrystals. It is also reasonable to expect that our strategy could be applied to other cocrystals with different donor and acceptor molecules, opening a new way for designing piezochromic luminescent materials. Moreover, it is possible to finely manipulate certain intermolecular interactions between donor and acceptor in a cocrystal by selecting the inserted nonemissive molecule with specialized functions (such as to form hydrogen bonding and promote molecular deformation by THF). This should also contribute to the design of new materials with desirable properties.

In summary, a strategy has been demonstrated to achieve piezochromic luminescent materials based on perylene–TCNB binary cocrystals by molecular insertion. We show that the insertion of THF, a nonemissive molecule, into perylene–TCNB cocrystal can manipulate intermolecular interactions between donor (perylene) and acceptor (TCNB) to produce desirable piezochromic luminescent properties. THF can selectively modify certain intermolecular interactions by forming hydrogen bonding with the acceptor and promoting molecular deformation of the donor. This leads to anomalous, simultaneous pressure-induced blue-shift and enhanced emission in the perylene–TCNB-based cocrystals, very different from the red-shift and quenched emission in compressing perylene–TCNB cocrystals and the other cocrystals reported. This strategy is efficient for other molecules acting as a nonemissive component in perylene–TCNB cocrystals, resulting in anomalous piezochromic luminescent behaviors.

## Methods

### Material source

Perylene (98%) and 1,2,4,5-tetracyanobenzene (TCNB, 97%) were purchased from Tokyo Chemical Industry Co., Ltd. (TCI). Tetrahydrofuran (THF, HPLC) was purchased from Sinopharm Chemical Reagent Co., Ltd. All of the chemicals were used as received without further purification.

### Material synthesis and crystal structure

The PTCs were obtained by a solvent evaporation method, which has been reported elsewhere^11,15,21,28,31^. In brief, equimolar quantities of perylene (25.2 mg) and TCNB (17.8 mg) were dispersed in 20 mL of THF with ultrasonication, and an orange-yellow solution was obtained. The blackish-green PTCs were obtained after evaporation of solvent from the solution after 3–4 days at ambient conditions^31^. The as-synthesized PTCs had the same structure as those grown from CH_2_Cl_2_^30^ (Cambridge Crystallographic Data Centre (CCDC) number, 1248027). The PTCs–THF were obtained by immersing PTCs in THF solvent sealed in a capillary tube or diamond anvil cell and had the same structure as that reported in Ref. ^31^ (CCDC number, 1847279), but with different cell parameters according to our structural optimization of the experiment by using VASP code. The X-ray diffraction patterns of PTCs and PTCs–THF were calculated by using Materials Studio. DFT calculations were performed to determine the crystal structures at different pressures.

### High-pressure generation

High-pressure experiments were performed in a diamond anvil cell (DAC). Samples were loaded into a 120-µm-diameter hole drilled in the T301 stainless-steel gasket. Pressure was calibrated by the fluorescence emission of ruby in the sample chamber. In the experiments for PL, UV–visible absorption, IR and Raman measurements of PTCs–THF, excessive THF was added in the sample chamber, which also acted as the pressure transmitting medium (PTM) for the high-pressure experiments. The possible effect of THF pressure medium in these experiments has also been clarified, which was different from the THF inserted into the cocrystals and could be well distinguished upon compression. Furthermore, considering that molecular crystals were relatively soft, no PTM has been used in the high-pressure XRD experiments. In the experiments of PTCs, KBr was used as PTM for the IR measurements, while no PTM was used in the PL and Raman measurements.

### In situ high-pressure experiments

PL measurements were performed on a Raman spectrometer equipped with CCD detector (Renishaw in Via) in the fluorescence mode. The excitation source, a 514-nm line of a Cobolt FandangoTM laser with the total power of 36.8 mW (100%), was used for PL measurements. The PL spectra of PTCs and PTCs–THF were collected with acquisition time of 10 s and under laser powers of 17.19 μW (0.05%) and 0.068 μW (0.0001%), respectively. The sanwa Laser Power Meter LP1 was used to measure the power from the confocal microscope to the diamond anvil. The diameter of the laser spot focused on the sample was ~2 µm. Raman measurements were performed by the same Raman spectrometer (Renishaw in Via) and the spectra were excited by the 514.5 nm line or a Renishaw laser with 830-nm line. UV–visible absorption spectra were collected using a home-built fluorescence microscope equipped with a deuterium–halogen light source and a Horiba Jobin Yvon iHR320 spectrometer. Infrared measurements were carried out using a Bruker Vertex 80 V spectrometer with liquid nitrogen-cooled MCT detector. In situ high-pressure X-ray diffraction experiments were performed at the Rigaku Synergy Custom FR-X (*λ* = 0.7093 Å). Ambient-pressure X-ray diffraction experiments were performed at the Rigaku MicroMax-007HF at (*λ* = 1.5418 Å).

### Computational details

Our calculations were performed using first-principles plane-wave pseudopotential density functional theory (DFT) as implemented in the VASP code^49^. The projected augmented wave (PAW) method was employed with the PAW potentials taken from the VASP library where *2s*^*2*^ *2p*^*2*^, *2s*^*2*^ *2p*^*3*^, and *2s*^*2*^ *2p*^*4*^ were treated as the valence electrons of C, N and O atoms, respectively. A cutoff energy of 520 eV and an appropriate Monkhorst–Pack k-mesh density of 2*π* × 0.03 Å^−1^ are chosen to ensure that the enthalpy calculations are well converged to less than 1 meV/atom. The generalized gradient approximation (GGA) Perdew–Burke–Ernzerhof (PBE) was used to describe the exchange-correlation interactions. The molecular orbitals of complexes were calculated using the B3LYP/6-31 G (d, p). We calculated the properties of PTCs–THF in the crystal phase by using the QM/MM method with a two-layer ONIOM approach. The central TCNB–perylene–THF was selected as the high layer and treated by using the QM method, while the surrounding molecules were chosen as the lower layer and simulated by using the MM method. We adopted M06-2X/6-31 G (d, p) to study for QM and a universal force field (UFF) was applied for MM, the electronic embedding was adopted to describe the coupling of the QM/MM interfaces. All the calculations above were carried out in the Gaussian 09 package^50^. The IR and Raman spectra were calculated by the CASTEP code in the Materials Studio package. The exchange and correlation of electrons were treated by the GGA with the PBE functional, and the OTFG norm conserving pseudopotentials were used for calculations.

## Supplementary information

Supporting Information

## Data Availability

Data that support the findings of this study are available from the corresponding author upon reasonable request. The .cif files for the PTCs–THF cocrystal structure reported in this study are deposited in the Cambridge Structural Database with CCDC number 2081895. Source data are provided with this paper.

## Source data

Source Data
