# Supplementary material for: Molecular insertion regulates the donor-acceptor interactions in cocrystals for the design of piezochromic luminescent materials
Source: Nat Commun. 2021 Jul 2;12:4084. doi: 10.1038/s41467-021-24381-5 (PMC8253821; doi:10.1038/s41467-021-24381-5)
Supplement: Supplementary file 1 — Supporting Information [file 41467_2021_24381_MOESM1_ESM.pdf]

Supporting Information

Molecular insertion regulates the donor-acceptor interactions in cocrystals for the design of piezochromic luminescent materials

Chunguang Zhai[1], Xiu Yin[1], Shifeng Niu[1], Mingguang Yao[*,1], Shuhe Hu[1], Jiajun Dong[1], Yuchen Shang[1], Zhigang Wang[2], Quanjun Li[1], Bertil Sundqvist[1,3] & Bingbing Liu[*,1]

[1] State Key Laboratory of Superhard Materials, College of Physics, Jilin University, Changchun 130012, China

[2] Institute of Atomic and Molecular Physics, Jilin University, Changchun 130012, China

[3] Department of Physics, Umeå University, SE-90187 Umeå, Sweden

Contacts:

yaomg@jlu.edu.cn;

liubb@jlu.edu.cn

Compound Name	**PTCs**	**PTCs-THF**
Space Group	**P 21/c**	**C 2/M**
Cell Lengths(Å)	a = 7.889	a = 13.205
	b = 30.004	b = 14.569
	c = 8.936	c = 7.9733
Cell Angles (°)	α = 90	α = 90
	β = 91.493	β = 97.083
	γ = 90	γ = 90
Cell Volume(Å^3)	2114.5	1522.2

Supplementary Table 1. Crystallographic data for PTCs and PTCs-THF obtained from our experiments.

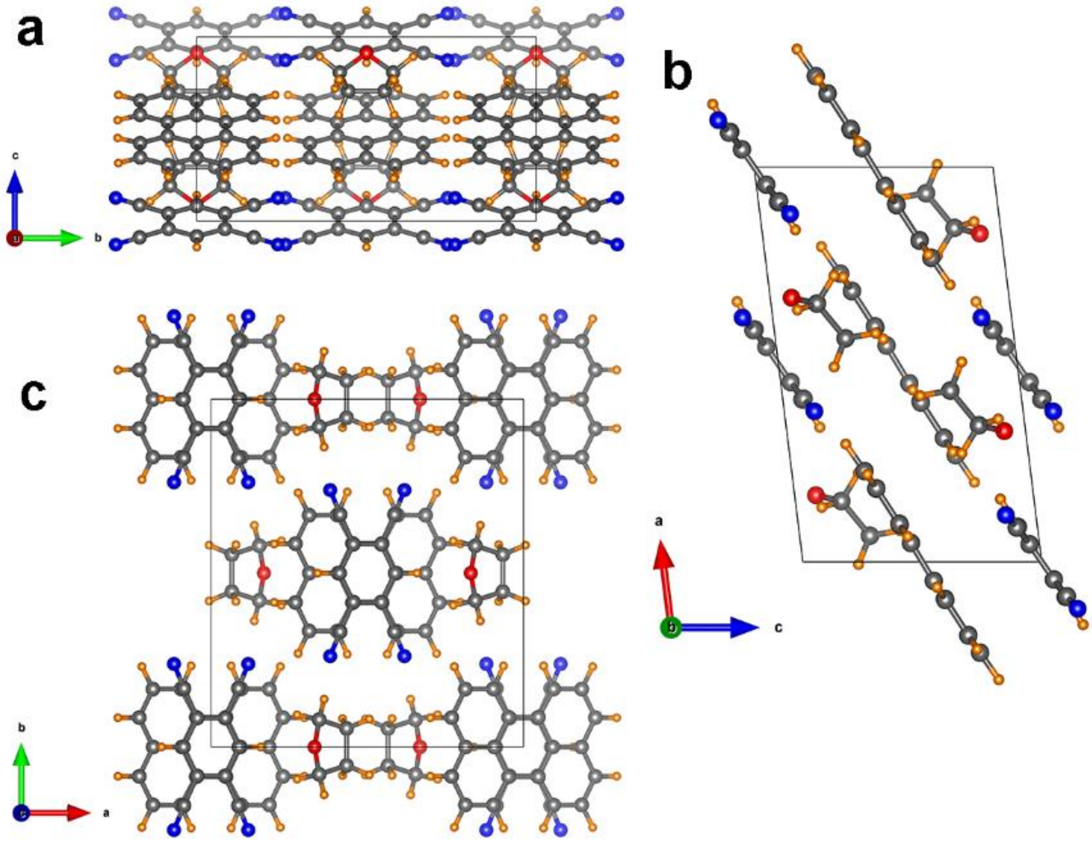

Supplementary Figure 1. Molecular packing of PTCs-THF perpendicular to the **a)** *a*-, **b)** *b*-, and **c)** *c*-axis.

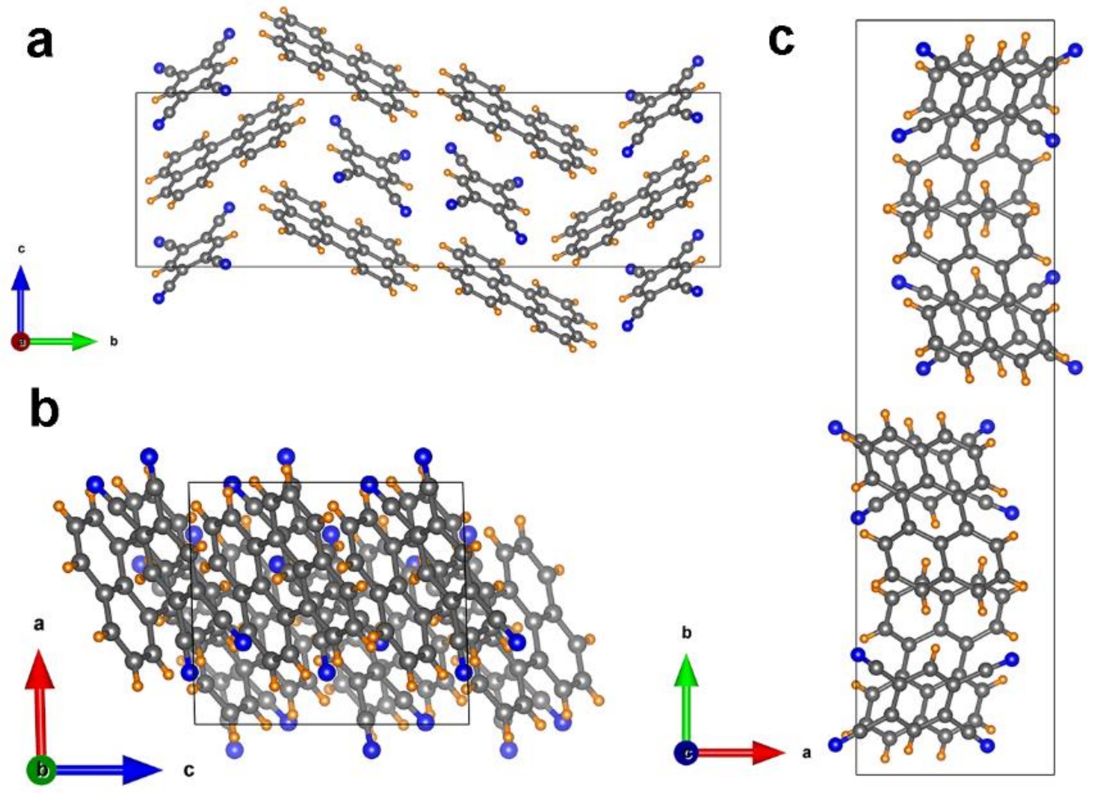

Supplementary Figure 2. Molecular packing of perylene-TCNB cocrystals (PTCs) perpendicular to the **a)** *a*-, **b)** *b*-, and **c)** *c*-axis.

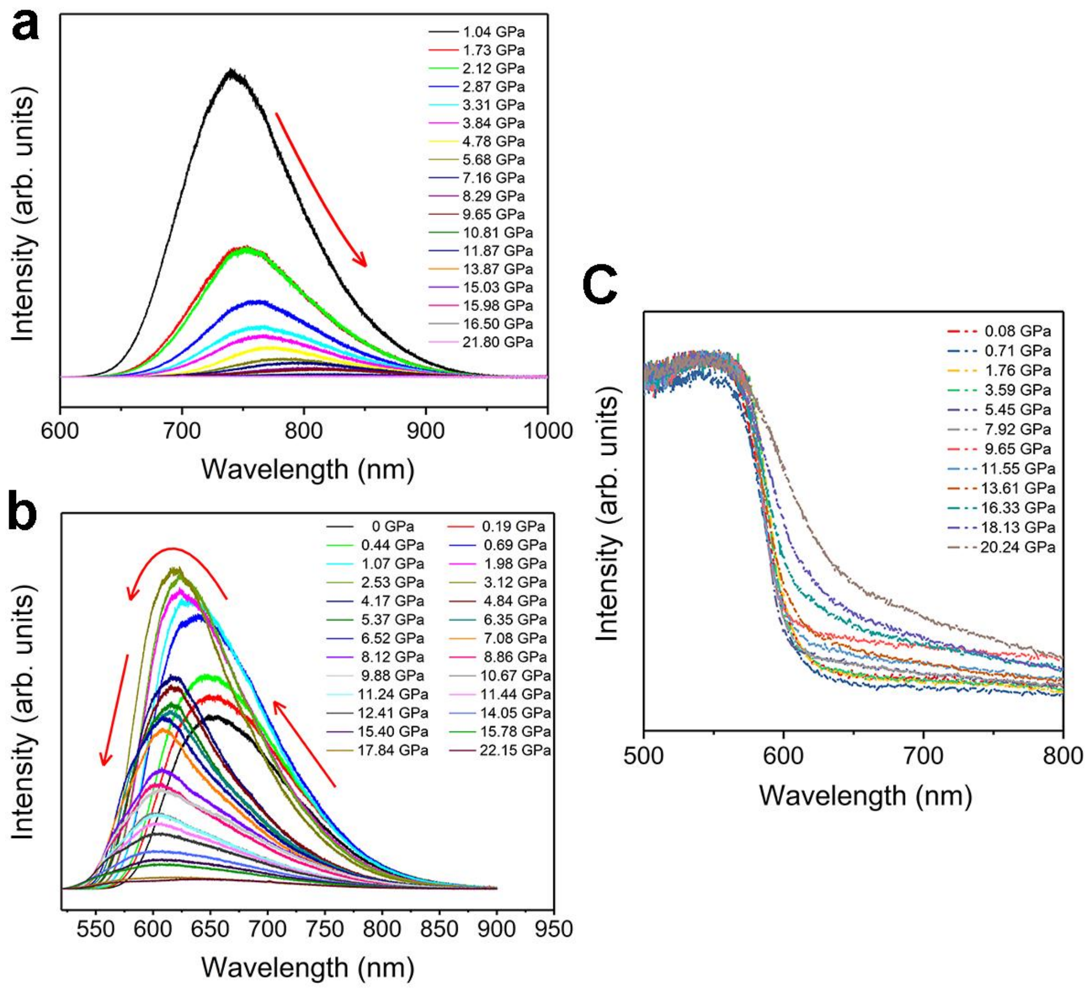

Supplementary Figure 3. a) PL spectra of PTCs under pressure up to 21.80 GPa. **b)** PL spectra of PTCs-THF up to 22.15 GPa. **c)** *In situ* UV-vis absorption spectra of PTCs-THF up to 20.24 GPa.

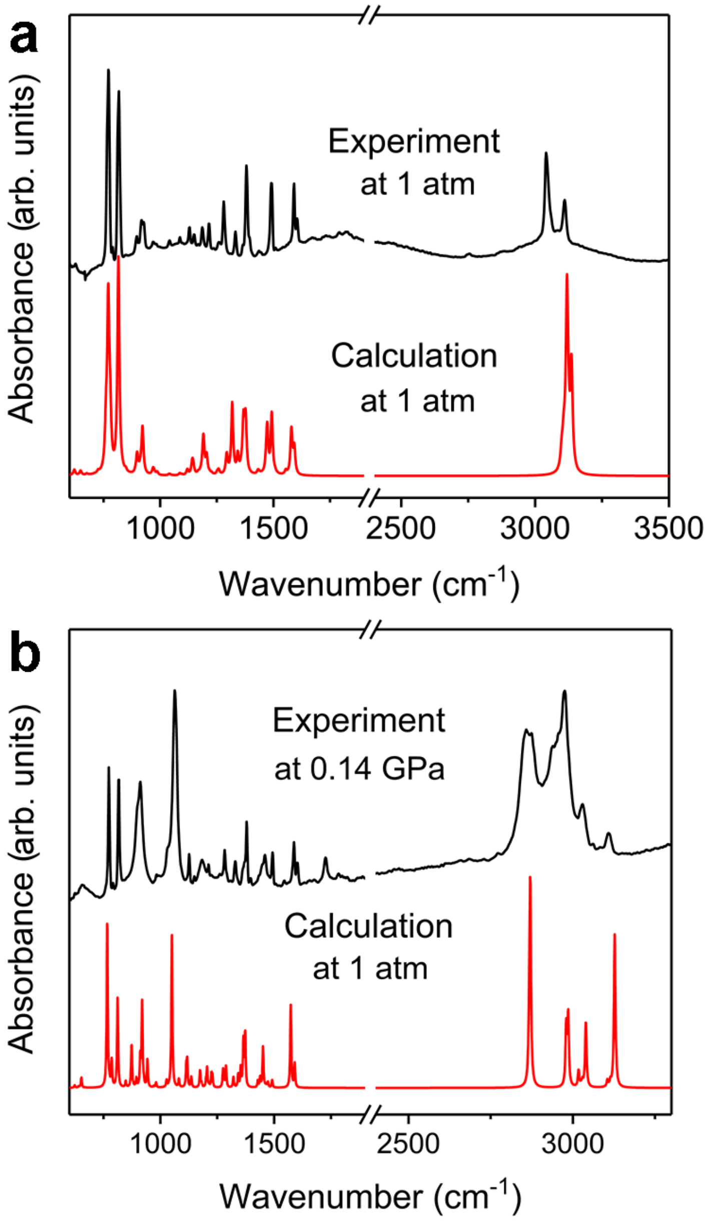

Supplementary Figure 4. a) Experimental (top) and calculated (bottom) IR spectra of PTCs. **b)** Experimental (top) and calculated (bottom) IR spectra of PTCs-THF.

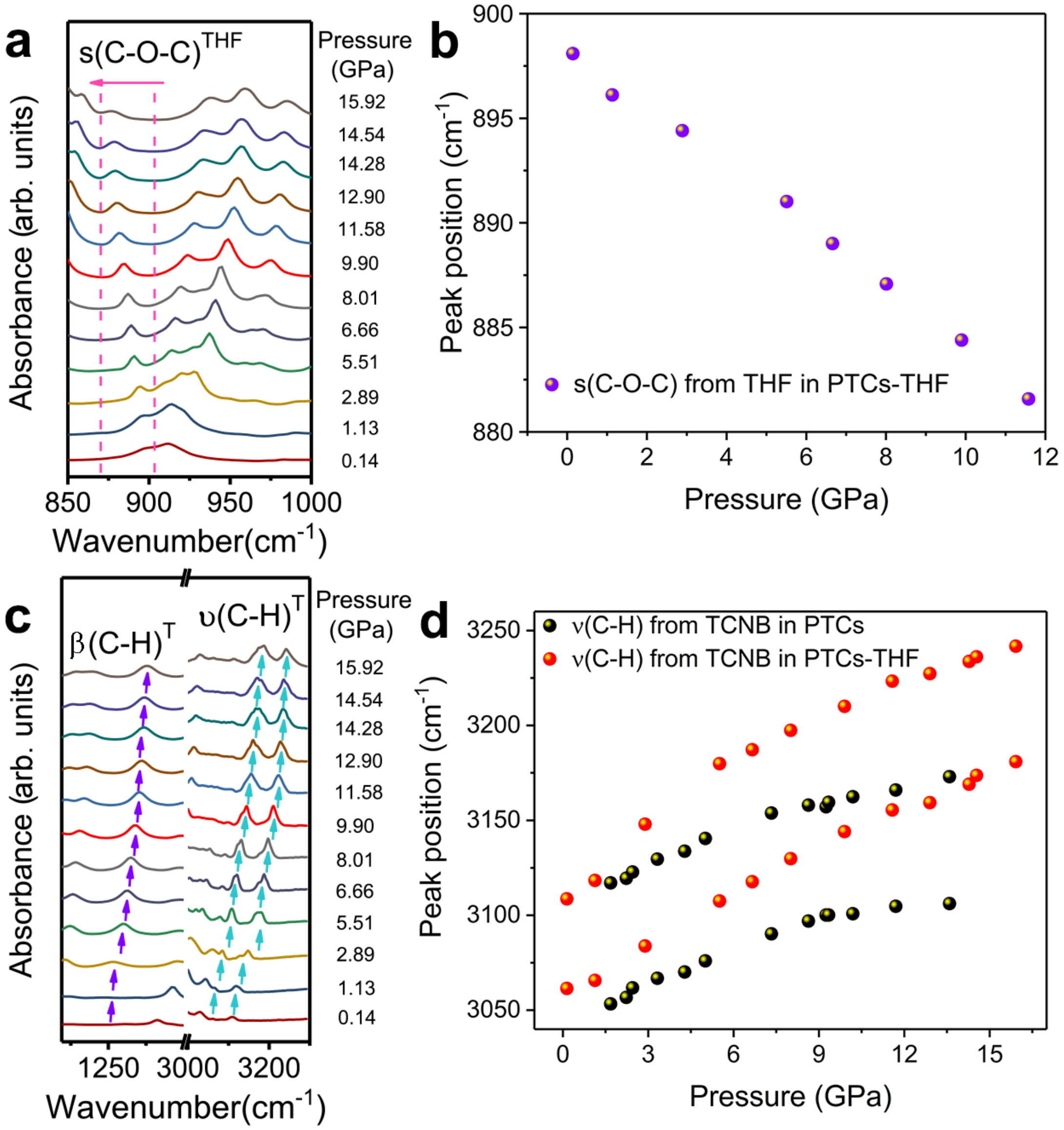

Supplementary Figure 5. The enlarged part of IR peaks and pressure dependence of the peak positions for some selected IR modes for PTCs-THF and PTCs. The enlarged part of IR peaks around **a)** 900 cm^{-1}, **c)** 1250 cm^{-1} and 3000 cm^{-1} of PTCs-THF. The pressure dependence of the peak position for **b)** C-O-C symmetrical stretching vibration s(C-O-C) from THF in PTCs-THF; **d)** C-H stretching vibration ν(C−H) from TCNB.

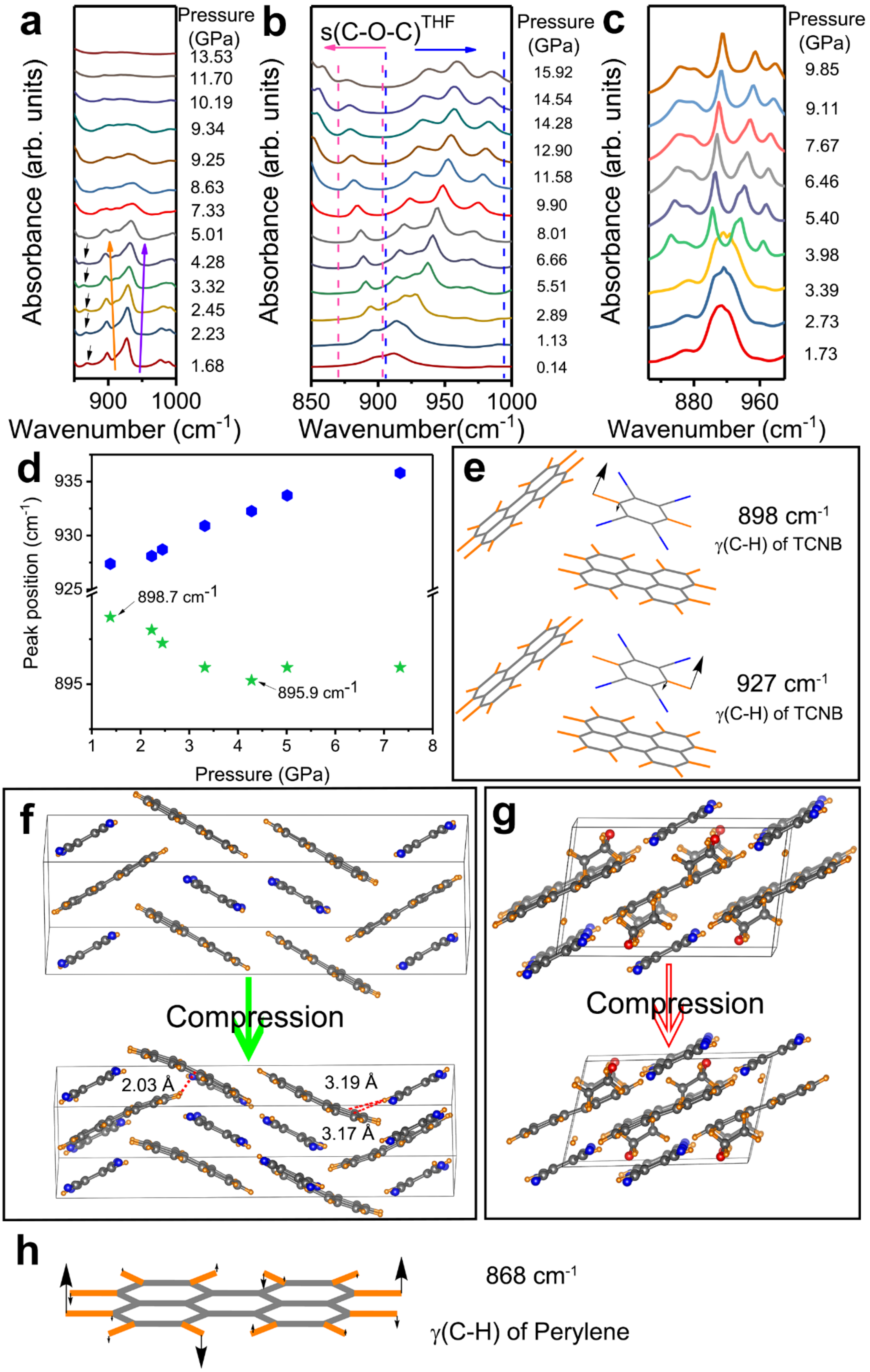
a
Pressure (GPa)
13.53
11.70
10.19
9.34
9.25
8.63
7.33
5.01
4.28
3.32
2.45
2.23
1.68
Absorbance (arb. units)
900
1000
Wavenumber (cm-1)
b
s(C-O-C)THF
Pressure (GPa)
15.92
14.54
14.28
12.90
11.58
9.90
8.01
6.66
5.51
2.89
1.13
0.14
Absorbance (arb. units)
850
900
950
1000
Wavenumber(cm-1)
c
Pressure (GPa)
9.85
9.11
7.67
6.46
5.40
3.98
3.39
2.73
1.73
Absorbance (arb. units)
880
960
Wavenumber (cm-1)
d
Peak position (cm-1)
935
930
925
895
898.7 cm-1
895.9 cm-1
1
2
3
4
5
6
7
8
Pressure (GPa)
e
898 cm-1
γ(C-H) of TCNB
927 cm-1
γ(C-H) of TCNB
f
Compression
2.03 Å
3.19 Å
3.17 Å
g
Compression
h
868 cm-1
γ(C-H) of Perylene

Supplementary Figure 6. The enlarged IR peaks around 900 cm^{-1} of **a)** PTCs, **b)** PTCs-THF and **c)** THF. **d)** The pressure dependence of the frequencies for C-H bending vibrations of TCNB. The sketch maps for the IR vibrations, **e)** the out-of-plane bending vibrations γ(C-H) of TCNB at 898 cm^{-1} and 927 cm^{-1}; **h)** the out-of-plane bending vibrations γ(C-H) of perylene at 868 cm^{-1}. The size of the arrow represents the amplitude of the corresponding vibration. The molecular packing of **f)** PTCs and **g)** PTCs-THF at 0 GPa (top) and 10 GPa (bottom) from calculations.

The red shift of the peaks observed in figure 4b (with no THF) upon pressure can be clearly seen in Supplementary Fig. 6a when we enlarged the IR peaks around 900 cm^{-1}. According to our assignment, the two peaks at 898 cm^{-1} and 927 cm^{-1} should be from the C-H out-of-plane vibration of TCNB (the corresponding vibrations are shown in Supplementary Fig. 6e). From the crystal structure of PTCs, we can see that the different frequencies of the two type of C-H vibrations are due to the different molecular stacking, that is, the C-H vibration facing to perylene is 898 cm^{-1}, in this case, C-H$\cdots\pi$ should form due to the suitable stacking of the two molecules (the C-H side of TCNB is close to perylene); in contrast, the vibration frequency of the other type of C-H vibration of TCNB has little possibility to form C-H$\cdots\pi$ interaction, which thus exhibits normal blue shift upon compression. Therefore, it is reasonable that in PTCs one of the C-H vibrations exhibits red shift upon

compression due to the weak C-H···π interactions[1](Supplementary Fig. 6f). On the other hand, similar effect could happen to the C-H vibrations of perylene. For example, the IR peak of C-H bending vibration at ~868.8 cm^{-1} has a weak red shift because of the formation of the weak C-H···π interaction (Supplementary Fig. 6a, h).

In the case of PTCs-THF, we can clearly see that one IR peak shows red shift upon compression when we enlarged the corresponding IR peaks around 900 cm^{-1} (Supplementary Fig. 6b). The IR peak at this frequency range (~900 cm^{-1}) could be from C-O-C vibration or C-H vibration (from the C-O-C vibration of THF and the C-H vibration of TCNB, Figure 4a), but several evidences strongly support that this red shift peak is most likely from C-O-C vibration in PTCs-THF. Firstly, the red shift peak was not from the C-H vibrations of THF, because the C-H vibrations of THF is at ~860 cm^{-1} and ~960 cm^{-1} (very weak intensity, Supplementary Fig. 6c), according to our experiments, calculations and previous literature. Secondly, due to the limitation of the molecular stacking (Supplementary Fig. 6g), there was little possibility to form C-H···π interactions in the PTCs-THF. It is worth noting that the C-H out-of-plane bending mode of TCNB only exhibits one vibration frequency, which is different from those of PTCs according to our theoretical calculations. Furthermore, in the case of PTCs the IR peak at 898cm^{-1} only shows ~3 cm^{-1} red shift (due to C-H…π interactions) upon compression (Supplementary Fig. 6d),

however, this IR peak exhibits a significant red shift of ~17 cm^{-1} in PTCs-THF, this should not be due to weak C-H…π interactions formed in PTCs-THF (Supplementary Fig. 5b). So, the red shift peak due to the C-H···π interactions can also be ruled out. Finally, the formation of blue-shifting hydrogen bond between C-H of TCNB and the C-O-C of THF (also evidenced by our theoretical calculation, see figure 6a) leads to the significant blue shift of C-H vibration of TCNB and the polarity increase of the C-H bond of TCNB (Supplementary Fig. 5c). This subsequently results in a significant red shift of the C-O-C vibration. Our comparison experiment on compressing pure THF can further support that the red shift of C-O-C vibration in PTCs-THF is due to the blue-shifting hydrogen bond effect, because we only observed a normal blueshift of C-O-C vibration in compressing pure THF as pressure increases (Supplementary Fig. 6c).

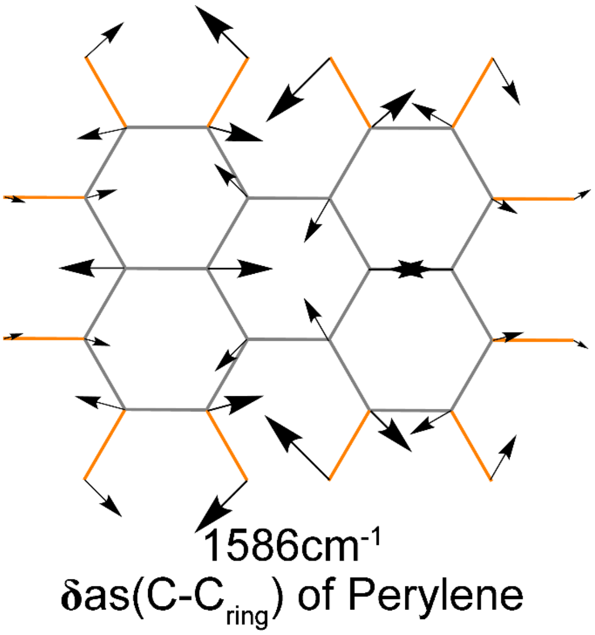

Supplementary Figure 7. The IR vibration at 1586 cm^{-1} is from the asymmetrical deformation vibrations δas(C-C_{ring}) of perylene. The size of the arrow represents the amplitude of the vibration.

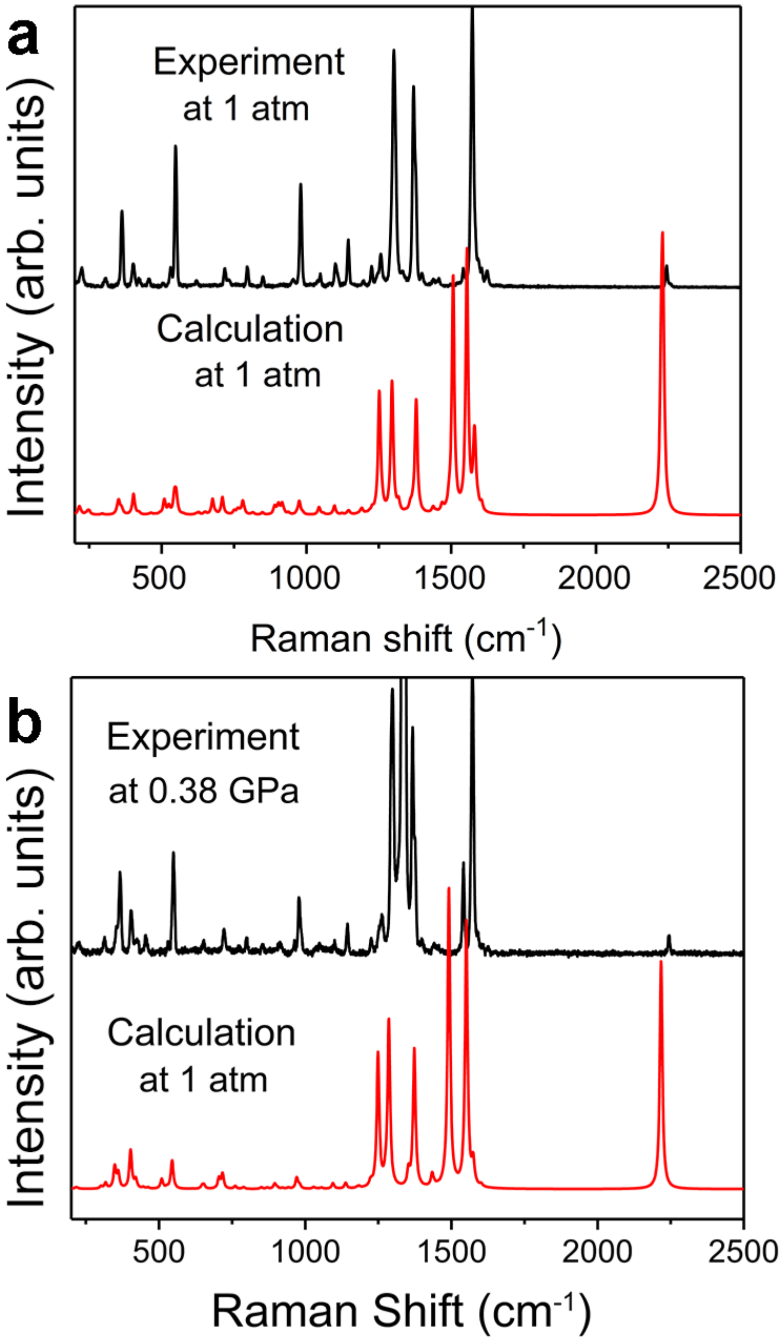

Supplementary Figure 8. a) Experimental (top) and calculated (bottom) Raman spectra of PTCs. **b)** Experimental (top) and calculated (bottom) Raman spectra of PTCs-THF.

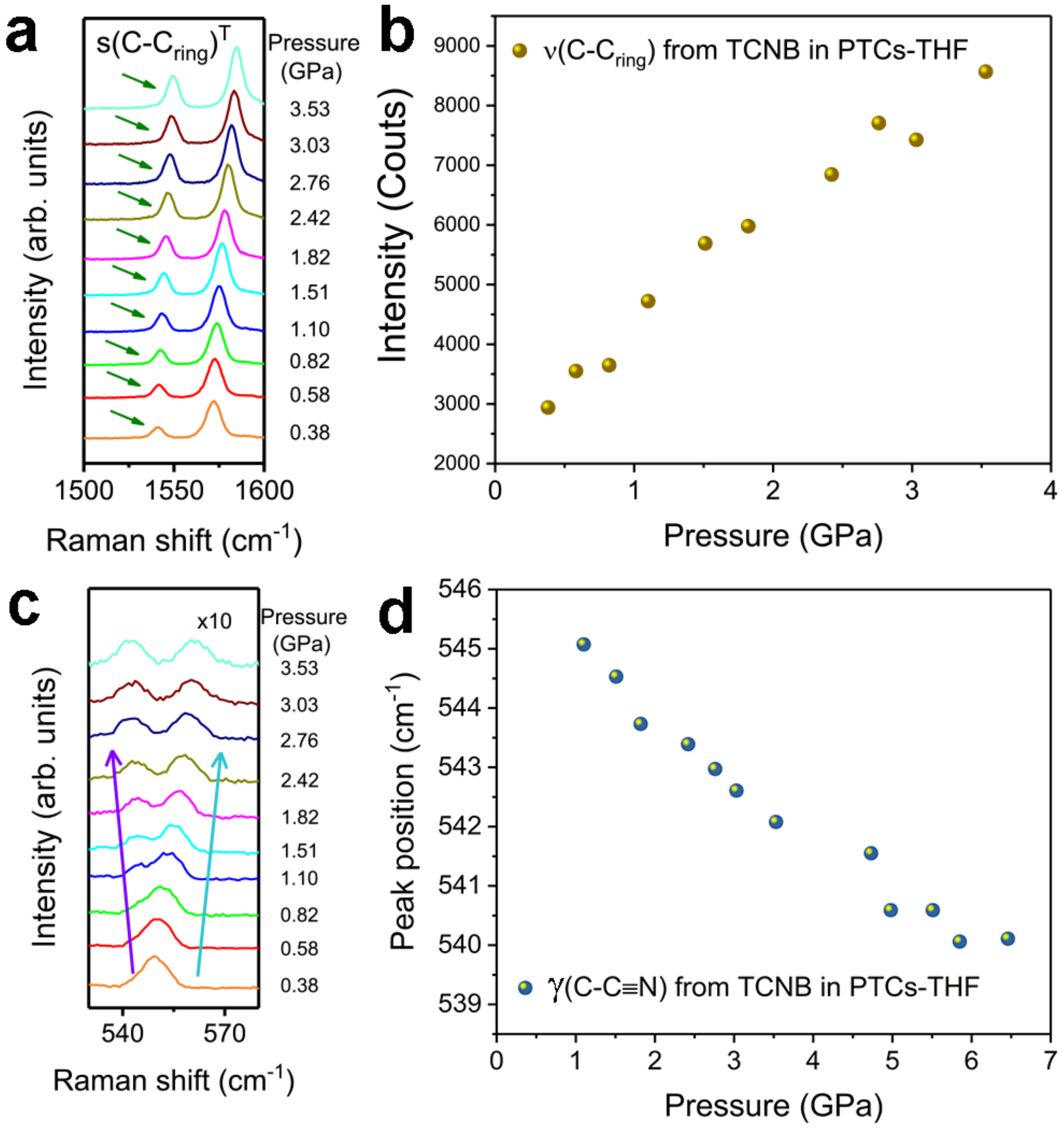

Supplementary Figure 9. The enlarged part of Raman peaks and pressure dependence of the peak positions and intensities for some selected Raman modes of PTCs-THF. The enlarged part of Raman peaks around **a)**1550 cm^{-1} and **c)**550 cm^{-1}. The dependence of **b)** peak intensity for the carbon ring stretching vibration ν(C-C_{ring}), and **d)** peak position for the C-C≡N out-plane bending vibration γ(C-C≡N) from TCNB.

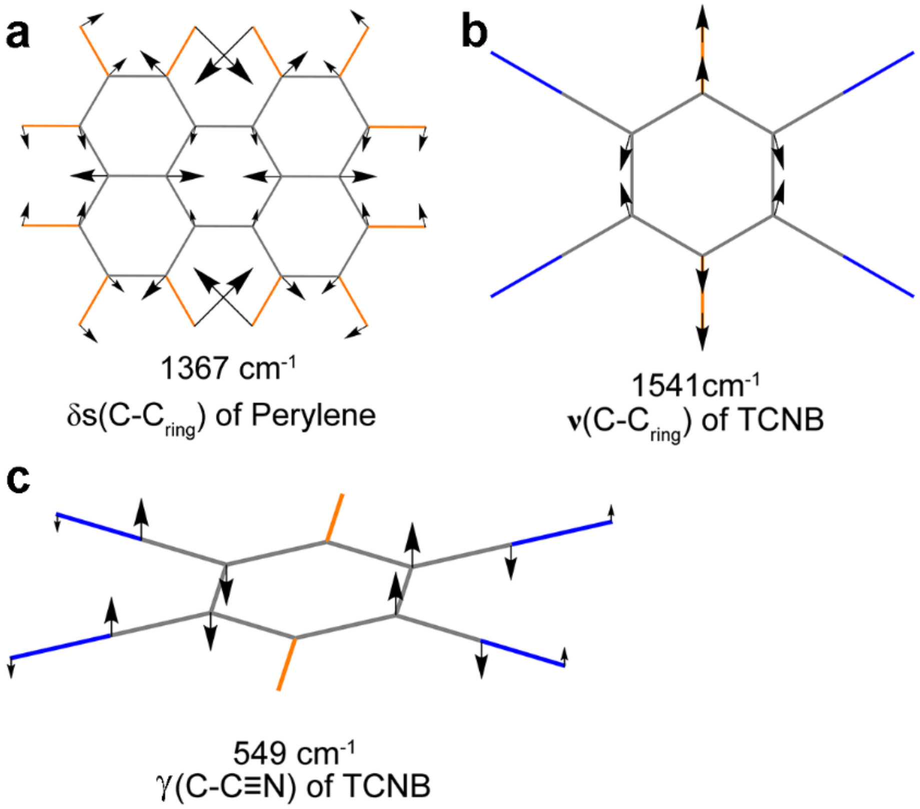

Supplementary Figure 10. The sketch maps for the Raman vibrations.

a) the symmetrical deformation vibrations $\delta s(C\text{-}C_{ring})$ of perylene at 1367 cm^{-1}; **b)** the carbon ring stretching vibration $\nu(C\text{-}C_{ring})$ of TCNB at 1541 cm^{-1}; **c)** out-of-plane bending vibration $\gamma(C\text{-}C{\equiv}N)$ of TCNB at 549 cm^{-1}. The size of the arrow represents the amplitude of the vibration.

**Approximate assignments**	**PTCs**			**PTCs-THF**		
	**Calculation**	**Experiment**		**Calculation**	**Experiment**	
		**IR**	**Raman**		**IR (0.14GPA)**	**Raman (0.38 GPa)**
symmetrical deformation (Perylene)				348.7		354.0
symmetrical deformation (Perylene)	360.1		362.9	360.6		366.5
symmetrical deformation (TCNB)	402.9		400.9	402.7		404.9
symmetrical deformation (Perylene)	545.0		548.5	544.6		549.1
out-of-plane C-C≡H bend (TCNB)	550.5		548.5	545.7		549.1
carbon ring stretch (TCNB)	710.0		717.9			
carbon ring symmetrical deformation (TCNB)				717.4		722.1
out-of-plane C-C-C-H bend (Perylene)	770.3	771.4		766.3	773.3	
out-of-plane C-C-C-H bend (Perylene)	778.6	790.6		785.9	792.6	
C-H_2 twisting vibration (THF)				785.9	792.6	
out-of-plane C-C-C-H bend (Perylene)	815.8	817.6		811.9	816.8	
out-of-plane C-H bend (TCNB)	898.0	896.7		873.4		
out-of-plane C-C-H bend (Perylene)	915.7	917.9		911.4		
C-O-C symmetrical stretch (THF)				919.5	898.5	
out-of-plane C-H bend (TCNB)	922.2	927.5				
C-H_2 twisting vibration (THF)				943.0		
out-of-plane C-H bend (Perylene)	968.7	968.8				
out-of-plane C-C-C-H bend (Perylene)	975.7		980.1	979.8		979.1
out-of-plane C-H bend (Perylene)	986.9	982.1		980.8	983.5	
C-H bend (THF)				1027.1	1031.4	
symmetrical deformation (TCNB)	1043.9	1041.3				
symmetrical deformation (TCNB)	1043.8		1048.2			
ring asymmetrical deformation (THF)				1050.5	1062.6	
in-plane C-H bend (Perylene)	1086.8	1087.6				
in-plane C-C-H bend (Perylene)	1097.0		1099.6	1094.7		1100.2
in-plane C-C-C-H bend (Perylene)	1119.9	1128.1		1117.8	1126.2	
in-plane C-C-H bend (Perylene)	1142.2		1144.8	1138.2		1144.6
in-plane C-C-C-H bend (Perylene)	1146.3	1149.3		1135.7	1149.3	
C-H bend (THF)				1174.2	1184.1	
in-plane C-C-H bend (Perylene)	1191.2	1185.9		1200.7		
C-H_2 twisting vibration (THF)				1205.3	1211.1	
in-plane C-C-C-H bend (Perylene)	1205.2	1215.2				
in-plane C-C-H bend (Perylene)	1227.5		1225.2	1222.1		1225.7
carbon ring breath (TCNB)	1251.8		1257.2	1249.1		1261.7
in-plane C-C-H bend (Perylene)	1293.3	1258.9		1288.2	1261.2	
carbon ring asymmetrical deformation (TCNB)	1318.1	1280.4		1320.2	1282.4	
in-plane C-C-H bend (Perylene)	1296.1		1302.5	1285.9		1298.3
symmetrical deformation (Perylene)	1342.5	1332.5		1341.7	1328.6	
C-H bend (THF)				1353.2	1361.5	
symmetrical deformation (Perylene)	1379.9		1370.1	1373.1		1367.5

asymmetrical deformation (Perylene)	1368.2	1367.2		1364.6	1369.2	
asymmetrical deformation (Perylene)	1376.8	1380.7		1372.5	1378.8	
asymmetrical deformation (Perylene)	1431.3	1436.5		1428.3	1432.8	
C-H bend (THF)				1438.5	1448.3	
C-H bend (THF)				1450.8	1459.8	
in-plane C-H bend (TCNB)	1472.7			1472.7	1475.3	
in-plane C-C-C-H bend (Perylene)	1492.5	1490.6		1491.2	1492.6	
carbon ring stretch (TCNB)	1506.8		1541.6	1491.3		1541.4
symmetrical deformation (Perylene)	1554.4		1573.1	1550.9		1572.2
asymmetrical deformation (Perylene)	1578.9	1590.9		1572.4	1587.1	
asymmetrical deformation (Perylene)	1592.1	1604.4		1590.2	1601.5	
carbon ring symmetrical deformation (TCNB)	1582.2		1605.8			
symmetrical deformation (Perylene)	1605.1		1623.6			
C≡N stretch (TCNB)	2230.5		2243.8	2216.9		2242.9
C-H stretch (THF)				2867.3<br>2870.4<br>2979.5<br>2985.6<br>3017.3<br>3039.4	2784<br>~3016	
C-H stretch (Perylene)	3106.8		3114.2		3027.6	
C-H stretch (TCNB)	3118.8	3041.1			3061.3	
C-H stretch (TCNB)	3135.7	3110.5		3127.2	3108.6	

Supplementary Table 2. Assignments of the experimentally observed IR and Raman modes for PTCs and PTCs-THF, according to our calculation.

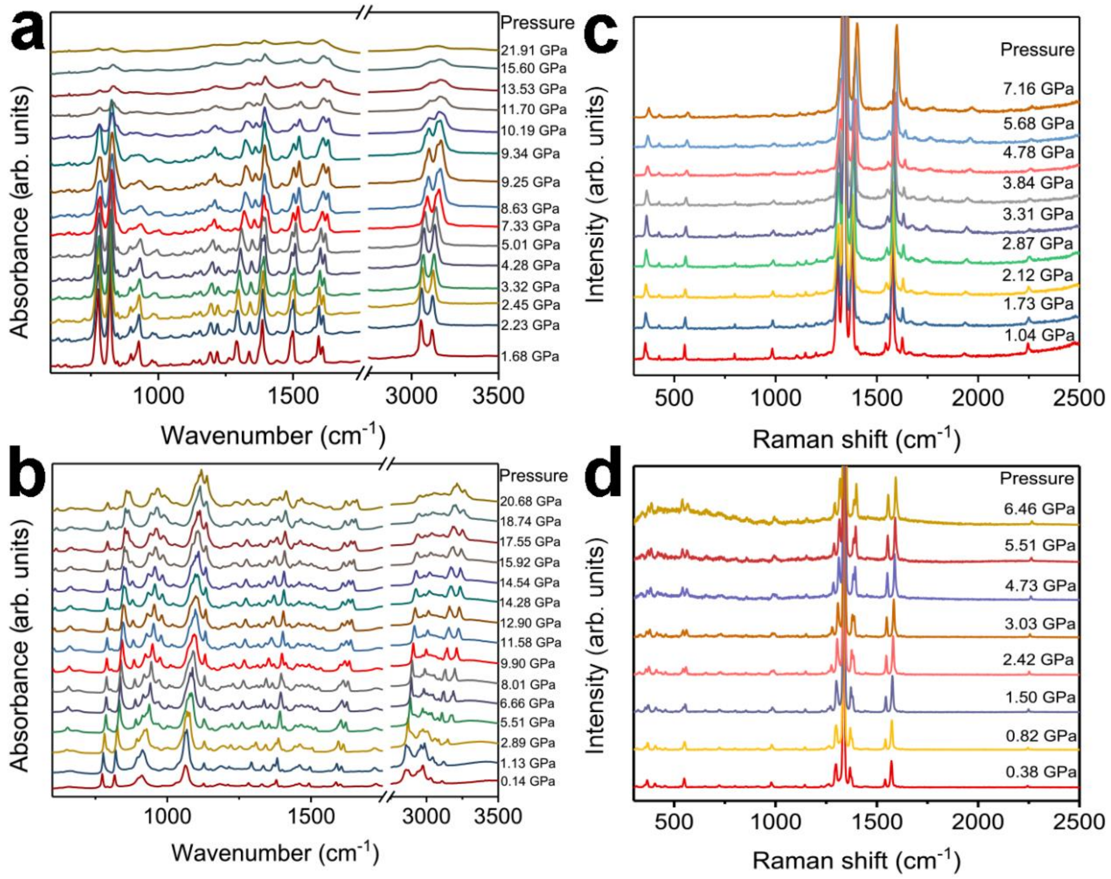

Supplementary Figure 11. IR and Raman spectra of PTCs and PTCs-THF. High-pressure IR spectra of **a)** PTCs from 1.68 GPa to 21.91 GPa and **b)** PTCs-THF from 0.14 GPa to 20.68 GPa. High-pressure Raman spectra of **c)** PTCs from 1.04 GPa to 7.16 GPa and **d)** PTCs-THF from 0.38 GPa to 6.46 GPa.

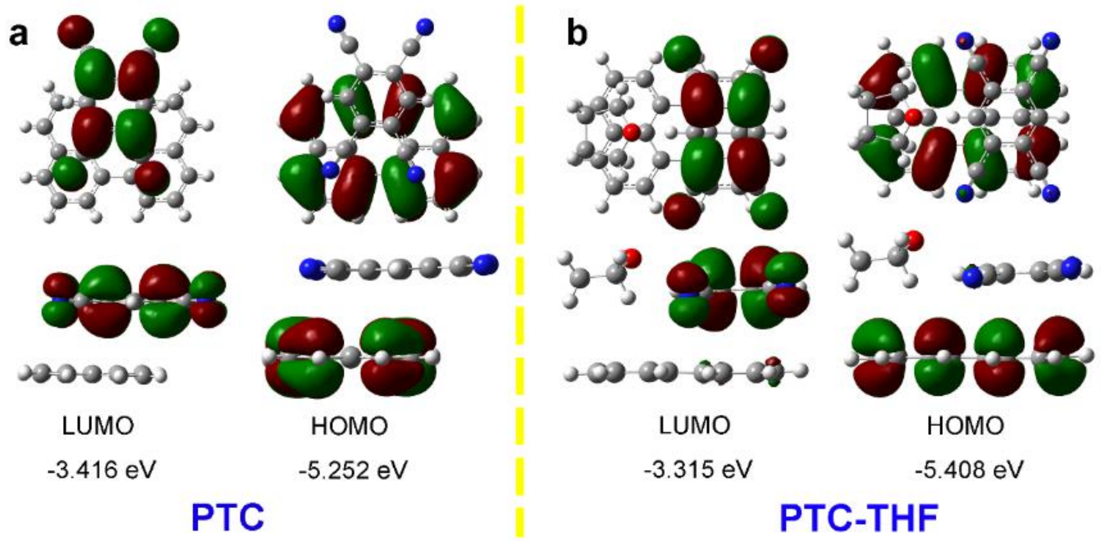

Supplementary Figure 12. Frontier orbital diagrams of the **a)** PTC and **b)** PTC-THF calculated by the density functional theory (DFT) method at B3LYP/6-31G (d, p).

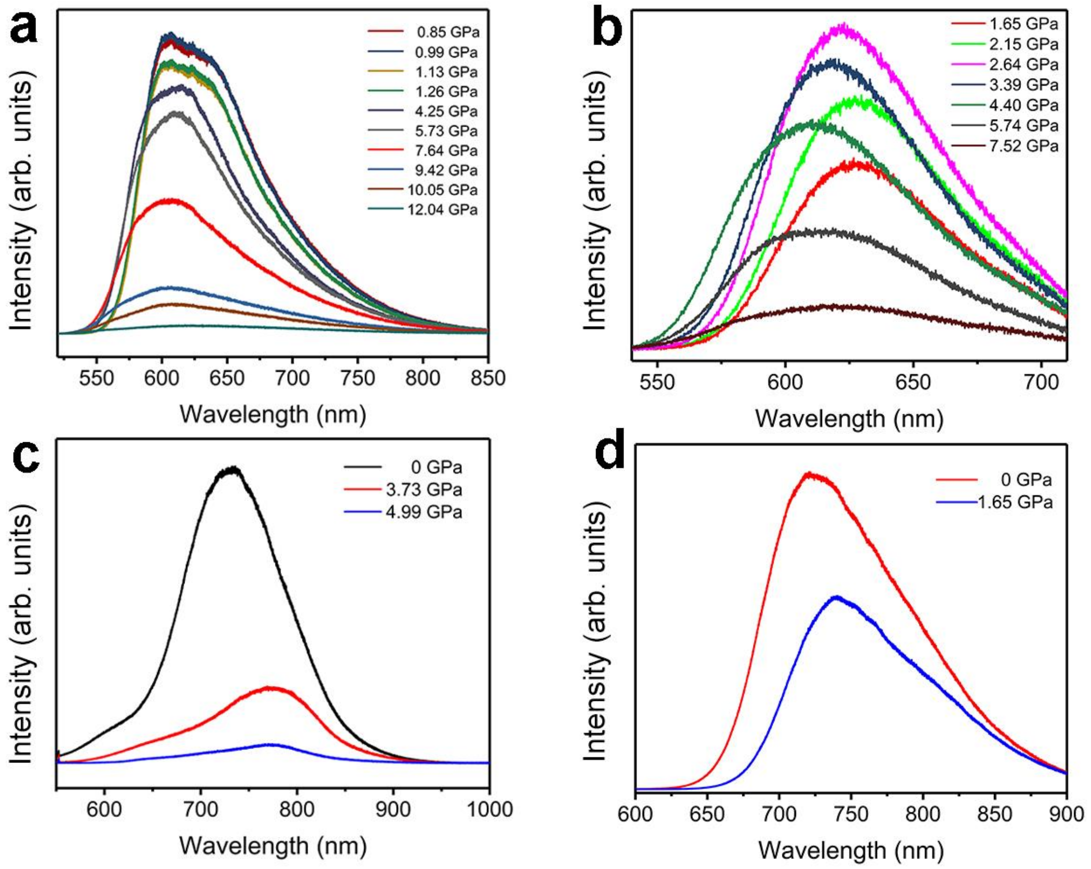

Supplementary Figure 13. PL spectra of perylene-TCNB cocrystals soaked in **a)** 1,4-dioxane, **b)** pyridine, **c)** CCl_4 and **d)** m-xylene.

Reference

1. Nishio M. CH/π hydrogen bonds in crystals. *CrystEngComm*. **6**, 130–158(2004).
